# Long non-coding RNAs in cutaneous melanoma: clinical perspectives

**DOI:** 10.18632/oncotarget.16478

**Published:** 2017-03-22

**Authors:** Eva Hulstaert, Lieve Brochez, Pieter-Jan Volders, Jo Vandesompele, Pieter Mestdagh

**Affiliations:** ^1^ Department of Dermatology, Ghent University Hospital, Ghent, Belgium; ^2^ Center for Medical Genetics, Ghent University, Ghent, Belgium; ^3^ Cancer Research Institute Ghent, Ghent University, Ghent, Belgium; ^4^ Bioinformatics Institute Ghent, Ghent University, Ghent, Belgium

**Keywords:** long non-coding RNA, melanoma, biomarkers, cancer therapy

## Abstract

Metastatic melanoma of the skin has a high mortality despite the recent introduction of targeted therapy and immunotherapy. Long non-coding RNAs (lncRNAs) are defined as transcripts of more than 200 nucleotides in length that lack protein-coding potential. There is growing evidence that lncRNAs play an important role in gene regulation, including oncogenesis. We present 13 lncRNA genes involved in the pathogenesis of cutaneous melanoma through a variety of pathways and molecular interactions. Some of these lncRNAs are possible biomarkers or therapeutic targets for malignant melanoma.

## NON-CODING RNA: FROM JUNK SEQUENCES TO REGULATORS OF DISEASE

Only 2% of the human genome sequence is translated into proteins.[1] Until recently, it was believed that only this small fraction of the genome contained relevant information. The remaining 98% of the human genome was referred to as useless ‘junk DNA’.[1–3] Recent advances in RNA-sequencing technology demonstrated that about 70% of this DNA can be transcribed into non-coding RNA (ncRNA) transcripts.[4] Unlike messenger RNAs, ncRNAs do not serve as a template for protein synthesis, but instead play a pivotal role in regulating protein-coding gene expression.[5] This mechanism seems more important in complex organisms, as suggested by the positive correlation between organism complexity and the proportion of the genome that is not coding for proteins.[6] There is increasing evidence that mutations and polymorphisms in the non-coding genome are associated with human disease.[7, 8] Small ncRNAs such as microRNAs (miRNAs, 18-24 nucleotides in length) are to be distinguished from long ncRNAs (lncRNAs, >200 nucleotides). MiRNAs are generally more sequence conserved across species and tissues and have been studied in more detail compared with the more recently discovered lncRNAs. While miRNAs negatively regulate gene expression, lncRNAs may induce or repress gene expression. As miRNAs have been studied for over a decade, medicines based on miRNAs are currently in clinical development whereas this is not yet the case for lncRNAs.[9, 10] Compared to miRNAs, the function of lncRNAs may be more specific in terms of species, tissue and tumor type. In addition, current research indicates that lncRNAs contribute to each of the six core hallmarks of cancer, as defined by Hanahan and Weinberg in 2000.[8, 11, 12] LncRNAs may thus be suitable both as biomarkers and as therapeutic targets.

## THE FUNCTION OF LNCRNAS

The functional evaluation of lncRNAs often relies on suppressing the activity of lncRNA (knockdown) and observing the resulting molecular phenotypes. Targeted knockdown of lncRNAs can be achieved using three different techniques: antisense oligonucleotides (ASO), RNA interference (RNAi) technology and more recently, the clustered regularly interspaced short palindromic repeats (CRISPR)/CRISPR-associated (Cas) system or the Crispr/Cas system.[13–16] All of these knockdown techniques can potentially be used for therapeutic purposes. LncRNAs exert their function through interactions with other cellular molecules, including chromatin, proteins and RNA.[8] LncRNAs can influence the transcription of genes as they bind and (re-)position transcription factors or proteins that help define the structure of chromatin.[17]

LncRNAs can repress gene expression through recruitment of the polycomb repressive complex 2 (PRC2) towards specific loci in the genome, resulting in tri-methylation of H3K27, marking transcriptionally silent chromatin. This mechanism was observed for HOTAIR, a lncRNA transcribed from the HOXC locus that interacts with PRC2.[18] Alternatively, lncRNAs can activate gene expression. This can occur through recruitment of the WDR5/MLL complex, resulting in tri-methylation of H3K4 [19] or through the recruitment of the Mediator complex, resulting in phosphorylation of H3S10 [20]. The lncRNA molecular functions include decoys or guides for transcription factors and chromatin modifiers; scaffolds for multi-protein complexes and sponges for miRNAs.[21] These functions are not mutually exclusive as several lncRNAs exhibit complex functions through the combination of multiple molecular mechanisms.[17] Cellular localization plays an important role in defining or restricting the function of lncRNAs. Many lncRNAs regulate nuclear events (chromatin interactions, transcriptional regulation and RNA processing), while cytoplasmic lncRNAs modulate mRNA stability or translation and influence cellular signaling cascades.[22] Apart from a functional classification, lncRNAs are often categorized on the basis of their genomic location and orientation compared to protein-coding genes (eg, sense, antisense, intronic of intergenic).[21, 23, 24]

## IDENTIFICATION OF LNCRNAS IN CUTANEOUS MELANOMA AND THEIR MOLECULAR INTERACTIONS

Malignant melanoma, a skin cancer arising from melanocytes, has an increasing incidence and a high mortality rate. While localized melanoma is effectively cured by surgical excision, the prognosis for metastatic melanoma remains poor despite recent therapeutic progress.[25] Oncogenic transformation of melanocytes to melanoma is the result of complex changes in multiple signaling pathways controlling cell cycle progression and apoptosis. A constitutive activation of the RAS/ERK/MAPK signaling pathway is frequently driven by activating mutations in BRAF or NRAS.[26] Activating mutations in the BRAF oncogene are present in 50 to 60% of melanomas, 90% of which produce an active mutant BRAF^V600E^ protein.[27–29] Recently developed BRAF inhibitors as well as immune checkpoint inhibitors can be highly effective in some patients leading to a tremendous survival gain in metastatic melanoma.[30, 31] However, a substantial proportion of patients either do not respond or develop therapy resistance.[32–35]

Recent studies indicate that lncRNAs are involved in the pathogenesis of malignant melanoma. Here, we review the growing body of literature on lncRNAs in melanoma and their possible clinical relevance. We identified thirteen human lncRNAs implicated in the pathogenesis of melanoma, as summarized in Table 1. The lncRNAs reported to be involved in the pathogenesis of cutaneous melanoma exert their function through different pathways and they interact with different molecular targets. A possible classification based on the currently known molecular interactions is provided below.

**Table 1:** Long non-coding RNAs in malignant cutaneous melanoma

lncRNA	expression in melanoma	cells/tissue analyzed	molecular interactions	putative or confirmed effect	potential clinical relevance	references
SPRY4-IT1	upregulated	- human melanoma cell lines, normal melanocytes and keratinocytes - 30 melanoma patient samples and normal skin samples - plasma samples from 70 patients and 79 healthy controls	binds to LPIN2	promotes melanoma cell proliferation, migration and invasion; inhibits apoptosis	prognosis (SPRY4-IT1 plasma level independent negative prognostic factor)	[40, 41, 91, 92]
BANCR	upregulated	- melanocytes +/− BRAF^V600E^ mutation- 2 BRAF^V600E^-mutant primary melanoma specimens - 103 primary melanoma samples and 12 melanocytic nevus samples - 5 human melanoma cell lines	interacts with chemokine CXCL11	promotes melanoma cell migration	- prognosis (positive correlation between BANCR expression and tumor stages, univariate survival analysis) - therapeutic target	[44, 45, 97]
			activates the ERK1/2 and JNK MAPK pathway	promotes melanoma cell proliferation		
HOTAIR	upregulated	- 3 primary melanoma samples and matched lymph node metastases - 9 benign nevi samples, 7 primary melanoma samples pT1a, 30 pairs of primary melanoma samples pT3/4 with corresponding metastases and 32 visceral metastases - serum samples of 34 patients - human metastatic melanoma cell line A375 - 63 melanoma samples and paired adjacent normal tissue - *in silico* analysis of Gene Expression Omnibus data	interacts with PRC2	promotes melanoma cell migration and invasion. promotes degradation of extracellular matrix (metastatic potential)	- therapeutic target in metastatic melanoma - prognosis (positive correlation between HOTAIR expression and tumor stages)	[62, 70, 72]}
MALAT1	upregulated	- human melanoma cell line A375 - 63 melanoma samples, paired adjacent normal tissue and lymph node metastases - 3 primary melanoma samples and matched lymph node metastases	interacts with splicing factors	MALAT1 knockdown hampers the migration of melanoma cells	prognosis (MALAT-1 as prognostic indicator of lymph node metastasis in melanoma)	[62, 67, 70]
			interacts with miR-183 and ITGB1	promotes melanoma cell proliferation		
UCA1	upregulated	- human melanoma cell lines (A375 and SK-MEL-2) - 3 primary melanoma samples and matched lymph node metastases - 19 metastatic melanoma tissue samples, 18 primary melanoma samples and 20 benign nevi samples	binds miR-507	promotes melanoma cell proliferation, migration and invasion	- prognosis (UCA-1 as prognostic indicator of lymph node metastasis in melanoma) - UCA1-miR-507-FOXM1 as an epigenetic therapeutic target	[62, 63]
CASC15	upregulated	- melanocyte cultures - 30 human melanoma cell lines - human melanoma xenograft mouse model of brain metastasis - 141 primary melanoma samples and metastatic tissue	unknown	promotes melanoma cell transition between proliferative and invasive states	prognosis (CASC15 level is an independent predictor of disease recurrence in patients with stage III melanoma)	[84]
ANRIL	upregulated	- human cutaneous melanoma cell line A375 and uveal melanoma cell line OM431 - 18 cutaneous, 10 uveal primary melanoma samples and 9 benign nevus/choroid/retina tissue samples	negatively regulates the expression of tumor suppressor proteins CDKN2A/2B	ANRIL knockdown hampers the migration of melanoma cells and reduces clonogenicity	- early diagnostic marker - therapeutic target	[52] [88, 89]
RMEL3	upregulated	- Human Expressed Sequence Tag and The Cancer Genome Atlas data - 20 human melanoma cell lines, including A375-SM- 19 nevi, 19 primary and 17 metastatic melanoma tissue samples	interferes with MAPK and PI3K signalling	RMEL3 knockdown decrease melanoma cell survival and proliferation	therapeutic target	[50, 51]
SNHG5	upregulated	- serum of 24 melanoma patients, 15 healthy subjects and 5 patients with spinocellular carcinoma - 36 primary melanoma samples and 4 nevi samples	unknown	unknown	- early diagnostic marker - monitoring of recurrence	[90]
SLNCR1	upregulated	- The Cancer Genome Atlas data- melanoma short-term cultures and fibroblast short-term cultures derived from the tumor	interacts with AR and BRN3A, resulting in the upregulation of MMP9	promotes melanoma cell invasion	- prognosis (high expression of SLNCR1 was associated with shorter overall survival) - therapeutic target	[75]
SAMMSON	upregulated	- melanoma patient derived tumor xenograft mouse model- The Cancer Genome Atlas data - 17 human melanoma cell lines	interacts with p32, a master regulator of mitochondrial homeostasis and metabolism	SAMMSON knockdown reduces clonogenicity	- diagnostic marker - therapeutic target	[58]
LLME23	upregulated	human melanoma cell lines (YUSAC, A2058, YU-SIT1, SKmel28)	binds with PSF. Suppresses proto-oncogene RAB23	LLME23-knockdown decreases colony forming ability of melanoma cells	therapeutic target	[60]
GAS5	downregulated	- human melanoma cell lines (HaCaT, A375, SK-Mel-28, SK-Mel-110 and M21) - tumor mouse model	possibly interacts with MMP2	inhibits the migration and invasion of melanoma cells	only preclinical research available	[80, 93]

### Regulation of apoptosis

Sprouty4-intronic transcript 1 (SPRY4-IT1 or SPRIGHTLY), a lncRNA derived from an intron of the SPRY4 gene, was the first lncRNA characterized in melanoma. Besides melanoma, SPRY4-IT1 has been reported to play an important role in esophageal squamous cell carcinoma,[36] prostate cancer,[37] glioma[38] and gastric cancer.[39] Mazar et al.[40] suggested that SPRY4-IT1 inhibits apoptosis via binding to LPIN2, thus altering lipid metabolism by avoiding cellular lipotoxicity. It has also been suggested that SPRY4-IT1 plays a role in post-transcriptional gene silencing based on its predicted secondary RNA structure with specific regulatory motifs.[41, 42] Others have suggested that SPRY4-IT1 manipulates the RAS/ERK pathway given its membership of the Sprouty family of RAS/ERK inhibitor proteins that prevent the formation of active GTP-RAS.[43] However, these hypotheses still need to be confirmed experimentally.

### LncRNAs associated with BRAF mutation

In an attempt to define the impact of oncogenic BRAF^V600E^ expression on the melanocyte transcriptome, Flockhart et al.[44] performed massively parallel RNA sequencing analysis on both normal melanocytes and two BRAF^V600E^-mutant human primary melanoma specimens and identified BRAF-activated non-coding RNA (BANCR). BANCR is overexpressed in BRAF^V600E^ melanoma cells. RNAi-dependent knockdown of BANCR inhibited melanoma cell migration by upregulating the chemokine CXCL11, a regulator of cell migration.[44] BANCR is also capable to promote melanoma proliferation by activating the ERK1/2 and JNK MAPK pathways both *in vitro* and *in vivo* in a mouse model.[45] More recently, upregulated BANCR expression has also been reported in retinoblastoma,[46] colorectal cancer [47] and gastric cancer.[48] In contrast, BANCR was downregulated in non small cell lung cancer (NSCLC).[49]

Based on the human Expressed Sequence Tag (EST) data and The Cancer Genome Atlas (TCGA) data, RMEL3 was identified as a lncRNA with a specific and increased expression in melanoma compared with normal tissues and melanocytes.[50, 51] A positive correlation between RMEL3 expression and the presence of the BRAF^V600E^ mutation was reported.[50] RMEL3 knockdown in melanoma cells of the A375-SM cell line resulted in the inactivation of the critical MAPK and PI3K signaling pathways.[51] BANCR and RMEL3 are two lncRNAs that seem to exert their effect through the MAPK pathway.

### Regulation of cell proliferation

Antisense non-coding RNA in the INK4 locus (ANRIL) was discovered after sequence-tagged site reverse transcriptase polymerase chain reaction (RT-PCR) based gene dose mapping of the entire INK4/ARF locus in a family with melanoma and neural system tumors.[52, 53] ANRIL is transcribed as a lncRNA in the antisense orientation relative to the P15/CDKN2B/INK4B-P16/CDKN2A/INK4A-P14/ARF cluster. The latter gene cluster encodes three tumor suppressor proteins and its transcription is an important barrier for tumor growth. CDKN2A mutations have been implicated in approximately 20-40% of familial melanomas.[54] ANRIL is thus believed to participate directly in epigenetic transcriptional repression.[53, 55] Apart from melanoma, ANRIL expression was upregulated in various other malignancies, such as colorectal cancer [56] and NSCLC.[57]

Recently, Survival Associated Mitochondrial Melanoma Specific Oncogenic RNA (SAMMSON) was identified by Leucci et al.[58, 59] as a novel melanoma-specific lncRNA that interferes with mitochondrial metabolism. Mechanistic studies indicated that SAMMSON interacts with P32, a master regulator of mitochondrial metabolism, to increase its mitochondrial targeting and promote oxidative phosphorylation.[58]

LLME23 seems to be involved in the colony forming ability of melanoma cells. LLME23 binds polypyrimidine tract-binding protein-associated splicing factor (PSF, also known as SFPQ).[60] PSF can exert a tumor-suppressor function by binding to the promoter of the proto-oncogene RAB23 that encodes a RAS-related small GTPase.[60]

Urothelial carcinoma associated 1 (UCA1) was originally detected in bladder transitional cell carcinoma where it is believed to promote invasion and cancer progression.[61] In melanoma, UCA1 was found to be upregulated in both primary and metastatic melanoma samples.[62, 63] UCA1 is believed to exert its oncogenic functions by acting as a miRNA sponge for miR-507. As a result, FOXM1, a target of miR-507, is downregulated upon UCA1 depletion in melanoma cell lines, resulting in the inhibition of cell proliferation.[63]

Metastasis-associated lung adenocarcinoma transcript 1 (MALAT1), also known as nuclear-enriched transcript 2 (NEAT2), was discovered as a prognostic marker for lung cancer metastasis.[64] Since its discovery in 2003, MALAT1 has been one of the most studied lncRNAs so far and is overexpressed in multiple carcinomas.[65, 66] In melanoma, MALAT1 may promote cell proliferation and invasion through a complex interaction with miR-183 and integrin β1 (ITGB1).[67]

### Regulation of metastasis

HOX antisense intergenic RNA (HOTAIR) is transcribed from the HOXC cluster and recruits PRC2 to specific target genes, leading to H3K27 trimethylation and epigenetic silencing of metastatic suppressor genes.[18, 68, 69] HOTAIR deregulation is associated with prometastatic activity in melanoma [70–72], but also in breast cancer, [69] cervical cancer [73] and pancreatic cancer.[74] HOTAIR has been suggested to stimulate the degradation of extracellular matrix through the activation of matrix metalloproteinases.[70]

SRA-like non-coding RNA (SLNCR1) was identified as a mediator of melanoma invasion.[75] SLNCR1 interacts with both the androgen receptor (AR) and the brain-specific homeobox protein 3A (BRN3A) and increases melanoma invasion by upregulating MMP9.[75] SLNCR1 is also upregulated in NSCLC.[76] The lncRNA growth arrest specific transcript 5 (GAS5) also alters the melanoma cell invasion through interaction with matrix metalloproteinases. GAS5 is presumed to have tumor suppressive potential and is downregulated in multiple malignancies.[77–80] Moreover, reduced GAS5 transcript levels in tumor tissue are associated with lymph node metastasis in cervical cancer [81] and hepatocellular cancer.[82] Chen et al.[80] detected the expression of GAS5 among five human melanoma cell lines. Reduced expression of GAS5 was observed in the SK-MEL-110 melanoma cell line using RT-PCR compared to the other cell lines (HaCaT, A375, SK-MEL-28 and M21). A375 cells expressed the highest GAS5 level.

### LncRNAs with unknown molecular mechanism

Small nucleolar RNA host gene 5 (SNHG5) is classified as a non-protein-coding multiple small nucleolar RNA host. The mechanism by which SNHG5 might interfere with the pathogenesis of malignant melanoma is not known yet. SNHG5 was also studied in gastric cancer, where SNHG5 was downregulated and associated with tumor, node and metastasis stage.[83]

Analysis of altered intergenic domains in metastatic melanoma by Lessard et al.[84] uncovered the cancer susceptibility candidate 15 (CASC15) as a lncRNA associated with metastatic cutaneous melanoma. The molecular interactions of CASC15 in melanoma is still unknown. In contrast to the oncogenic properties of CASC15 in melanoma, in neuroblastoma, another neural crest derived tumor, CASC15 has been described as a tumor suppressor lncRNA.[85] This points at context specific functions of lncRNAs, as documented previously for microRNAs.[86]

## LNCRNAS AS BIOMARKERS FOR CUTANEOUS MELANOMA

Traditionally, diagnosis of malignancies is based on tissue samples containing tumor cells. Expression levels of specific lncRNAs in tissue may become an additional tool in the pathologists’ armamentarium for difficult to diagnose cases.[87] Furthermore, sensitive molecular detection methods enable the identification of tumor-derived nucleic acids in serum or plasma. Such liquid biopsies can also be interrogated for lncRNAs. LncRNAs with overexpression levels reflected in serum or plasma, are therefore easily accessible candidate biomarkers for early tumor detection or disease progression.

All but one (GAS5) of 13 lncRNAs reviewed are upregulated in melanoma compared with normal tissue, and three lncRNAs are suggested to be melanoma specific (RMEL3, SAMMSON, LLME23). GAS5 is downregulated in melanoma and in multiple other malignancies.[77–80]

The expression levels of ANRIL [52, 88, 89] and SNHG5 [90] in primary melanoma samples have so far been compared with normal tissue only and have not yet been studied in relation to melanoma stage. ANRIL was overexpressed in 18 cutaneous and 10 uveal primary melanoma samples compared with 9 healthy tissue samples including benign nevi, choroid and retina tissue.[88]

The serum SNHG5 levels were significantly higher in 24 patients with melanoma compared to 15 healthy subjects and 5 patients with squamous cell carcinoma (SCC). In five patients with melanoma, the serum levels of SNHG5 were assessed before and after surgery. A significant decrease of the SNHG5 serum levels was observed after surgery, and in two of the five patients the serum levels increased at the time of recurrence. The expression of SNHG5 was also assessed in tissue samples of 36 primary melanoma and in four nevi. No significant differences in the SNHG5 level between both tissue types was observed.[90]

Below we group the findings of relevance for biomarker development into prognostic markers based on serum or plasma samples, prognostic markers based on tissue samples, and markers for lymph node involvement. In case lncRNAs have been investigated for their clinical relevance in melanoma tissue as well as in blood samples, we present the findings under serum or plasma markers.

### Prognostic markers in serum or plasma

The prognostic value of SPRY4-IT1 [91] and HOTAIR [72] was demonstrated in plasma and serum. Plasma levels of SPRY4-IT1 were increased in 70 melanoma patients compared with 79 healthy controls.[91] In addition, multivariate survival analysis revealed that a high SPRY4-IT1 expression level in plasma is an independent negative prognostic factor for overall survival in melanoma patients. Cantile et al.[72] recently reported a progressive increase in expression levels of HOTAIR in tumor tissue as well as in serum from pT1 stage to pT4 stage. Tang et al.[70] reported that HOTAIR is significantly overexpressed in metastatic lymph nodes compared with matched primary melanomas. Data mining of publicly available gene expression data revealed a higher HOTAIR expression level in melanomas compared with non-tumor tissues. This is in contrast with the findings of Tian et al.[62] who found similar HOTAIR expression levels in 63 melanoma samples and paired adjacent normal tissue.

### Prognostic markers in melanoma tissue

BANCR[45] and SLNCR1 [75] expression levels in primary melanoma samples were associated with lower survival rates. Li et al.[45] showed that the expression of BANCR was higher in 103 samples of primary melanoma compared to 12 melanocytic nevus samples. Furthermore, BANCR expression increased with clinical stages of malignant melanoma. An analysis of 72 patients revealed that the overall survival rates of patients with a high expression of BANCR in the primary tumor was lower than that of patients with a low expression of BANCR. [45] The clinical relevance of SLNCR1 was analyzed based on TCGA data. SLNCR1 expression was assessed across 150 randomly selected human melanoma tumors and high expression of SLNCR1 appeared to be associated with shorter overall survival.[75]

CASC15 is a marker of relapse in stage III melanoma patients.[84] The correlation of CASC15 expression level with shorter survival was based on RNA *in situ* hybridization in 141 formalin-fixed paraffin-embedded lymph node metastasis specimens.

### LncRNA as a marker of lymph node metastasis

MALAT1 [62] and UCA1 [63] are upregulated in melanoma and both may indicate lymph node metastasis in melanoma. The expression of MALAT1 in 63 lymph node metastatic tissue samples was higher than in paired primary melanomas. In addition, patients with lymph node metastasis exhibited a higher expression of UCA1 in the primary tumor than those without lymph node metastasis.[62] However, no significant difference in MALAT1 nor UCA1 expression was detected in (only) three pairs of primary melanoma and matched lymph node metastatic tissues by Tang et al.[70]

## LNCRNAS AS THERAPEUTIC TARGETS IN CUTANEOUS MELANOMA

From the 13 lncRNAs that are reviewed here, one has an unknown function in oncogenesis (SNHG5), one is regarded as a tumor-suppressor (GAS5) and 11 have pro-oncogenic properties in melanoma. Selective knockdown of these targets is a promising therapeutic strategy, especially if the expression is tumor specific as this would avoid unwanted effects in normal tissues. Most lncRNAs have been studied through RNA-interference mediated knock-down, two were also studies through ectopic overexpression. Of note, the latter will only reveal functions *in trans*, as the *cis-regulatory* lncRNA function requires expression from the endogenous locus in the genome.

### Knockdown of lncRNAs with oncogenic properties

RNAi-dependent knockdown of SPRY4-IT1 in the melanoma cell lines WM1552C and A375 prevented cell growth and resulted in decreased invasion and the induction of apoptosis.[40, 41, 77, 91, 92] Knockdown of HOTAIR in the human metastatic melanoma cell line A375 was shown to inhibit the motility and invasiveness of melanoma cells and resulted in a reduced degradation of the extracellular matrix.[70] *In vitro* studies using melanoma cell line A375 revealed that MALAT1 knockdown resulted in reduced cell migration.[62] The underlying mechanism by which MALAT1 affects melanoma cell migration has not yet been elucidated. Knockdown of UCA1 in A375 and SK-MEL-2 cell lines decreased the number of invasive melanoma cells and increased the fraction of G0/G1 phase cells.[63] In addition, *in vitro* studies by Tian et al.[62] revealed that A375 cells with reduced expression of UCA1 lncRNA, migrated less effectively through a transwell assay. Therefore, overexpression of UCA1 in A375 melanoma cells could promote cell migration.[62] Knockdown of CASC15 revealed that CASC15 regulates melanoma cell phenotype switching between proliferative and invasive states.[84] CASC15 knockdown in melanoma cell lines was associated with downregulation of the master regulator transcription factors MITF and SOX10 and concomitant upregulation of invasive signature genes.[84] ANRIL knockdown in the A375 cells led to an increase of the tumor suppressor proteins CDKN2A and CDKN2B; a decrease of the metastatic ability of melanoma cells in both a transwell and a wound-healing assay; and a reduction of clonogenicity in a colony formation assay.[88] Knockdown of the most prevalent isoform of SLNCR1 in the melanoma short-term culture WM1976 decreased melanoma invasion without affecting cell motility and proliferation.

SAMMSON, RMEL3 and LLME23 are pro-oncogenic and are exclusively upregulated in melanoma cells.[50, 51, 58, 60] SAMMSON functions as a lineage survival oncogene. Lineage survival oncogenes are characterized by a high lineage-specific expression, are upregulated in tumor cells derived from that lineage, and are required for their survival. ASO- and RNAi-mediated knockdown of SAMMSON reduced clonogenicity of all SAMMSON-expressing melanoma cultures and induced apoptosis of the melanoma cells, irrespective of BRAF, NRAS or TP53 status.

*In vivo*, the intravenous administration of a SAMMSON-specific antisense oligonucleotide in a melanoma patient derived tumor xenograft (PDTX) significantly reduced tumor growth. The combined administration of a SAMMSON-specific ASO and dabrafenib, a BRAF inhibitor, induced apoptosis in the PDTX model, whereas administration of dabrafenib alone only inhibited tumor growth. In addition, the combination of BRAF inhibitors and SAMMSON-targeting ASOs did not show signs of toxicity in contrast to combination treatment with BRAF and MEK inhibitors.[58]

RMEL3 knockdown in BRAF^V600E^ melanoma cells of the A375-SM cell line reduced colony formation and caused an accumulation of cells in the G1 phase together with an increase in apoptosis. A reverse phase protein array analysis was carried out to assess the effect of RMEL3 knockdown in A375-SM cells. A decrease in protein levels of AKT1 (pAKT), BRAF, RB1 (pRB) and CCNB1 (cyclin B1) together with an increase in PTEN and the G1/S cyclin-dependent kinase inhibitors CDKN1A and CDKN1B was observed, which reflects an inactivation of the critical MAPK and PI3K signaling pathways.[51]

Overexpression of LLME23 in YUSAC melanoma cells was induced through transfection with a LLME23-encoding plasmid and resulted in the upregulation of RAB23. In contrast, knockdown of LLME23 lead to a decreased expression of RAB23. LLME23-depleted YUSAC cells showed a significant decrease in their colony forming ability in soft-agar and the same cells demonstrated a 75% smaller tumor volume 38 days after injection into nude mice. LLME23 appears to be exclusively expressed in human melanoma cell lines. The latter feature might indicate an association of LLME23 expression with the etiology of human melanoma, however future research in patient samples should confirm this statement.[60]

### Overexpression of lncRNAs with a tumor suppressor function

GAS5 is the only reported lncRNA acting as tumor suppressor in melanoma. Overexpression of GAS5 in the SK-MEL-110 melanoma cell line reduced the migration and invasiveness and lead to a decrease in MMP2 expression, a protein specifically involved in type IV collagen degradation. Knockdown of GAS5 in A375 cell line facilitated wound healing in a wound healing assay and increased the proteolytic potential.[80] GAS5 administered to nude mice significantly inhibited tumor growth of GAS5 expressing melanoma cells.[80, 93–95] Further research is awaited to explore if enhancing GAS5 in melanoma cells could have therapeutic value.

## CONCLUDING REMARKS AND FUTURE DIRECTIONS

We reviewed lncRNA biology with particular attention for discoveries of relevance in the management of melanoma. LncRNA research evolves quickly. The current state of knowledge indicates that 13 lncRNA genes are involved in the pathogenesis of melanoma. Their cell or tumor type specific expression pattern makes them good candidate diagnostic markers or therapeutic targets.

Besides the need for novel, more effective and less toxic therapeutic strategies, there is a need for markers to predict or monitor the response to the new and expensive metastatic melanoma treatments. Further studies are needed to explore the role of lncRNAs for this purpose.

A limitation of the available evidence is that for some lncRNAs the reported results are conflicting. For example, Tian et al.[62] did not find a significant difference between the HOTAIR expression in primary melanoma samples and adjacent normal, while a higher HOTAIR expression level was detected in melanomas compared with nontumor tissues through data-mining of publicly available gene expression data.[70] Additional limitations may be related to the experimental methods and study design. Often the number of samples is relatively low to draw reliable conclusions or the selected cell lines are only representative for a subset of melanomas observed in patients. Results based on underpowered experiments need independent confirmation in sufficiently powered experiments before science can move forward.

In conclusion, the case of melanoma illustrates the rapidly growing understanding of the role of lncRNAs in oncology. In the short term, and after sufficient clinical validation, the use of one or multiple lncRNAs for melanoma biomarker applications can reasonably be expected. This strategy has already been implemented in prostate cancer with the introduction of lncRNA PCA3 as a commercial urine-based diagnostic test.[96] Even more important might be the therapeutic offspring of this research. Following the example of the miRNAs, blocking lncRNAs may also have therapeutic potential. The high tumor specificity of some of the lncRNAs might be a key success factor in this endeavor.

## References

[1] Lander ES, Linton LM, Birren B, Nusbaum C, Zody MC, Baldwin J, Devon K, Dewar K, Doyle M, FitzHugh W, Funke R, Gage D, Harris K (2001). Initial sequencing and analysis of the human genome. Nature.

[2] Volders PJ, Helsens K, Wang X, Menten B, Martens L, Gevaert K, Vandesompele J, Mestdagh P (2013). LNCipedia: a database for annotated human lncRNA transcript sequences and structures. Nucleic Acids Res.

[3] Ling H, Vincent K, Pichler M, Fodde R, Berindan-Neagoe I, Slack FJ, Calin GA (2015). Junk DNA and the long non-coding RNA twist in cancer genetics. Oncogene.

[4] Djebali S, Davis CA, Merkel A, Dobin A, Lassmann T, Mortazavi A, Tanzer A, Lagarde J, Lin W, Schlesinger F, Xue C, Marinov GK, Khatun J (2012). Landscape of transcription in human cells. Nature.

[5] Qiu MT, Hu JW, Yin R, Xu L (2013). Long noncoding RNA: an emerging paradigm of cancer research. Tumour Biol.

[6] Liu G, Mattick JS, Taft RJ (2013). A meta-analysis of the genomic and transcriptomic composition of complex life. Cell Cycle.

[7] Maurano MT, Humbert R, Rynes E, Thurman RE, Haugen E, Wang H, Reynolds AP, Sandstrom R, Qu H, Brody J, Shafer A, Neri F, Lee K (2012). Systematic localization of common disease-associated variation in regulatory DNA. Science.

[8] Schmitt AM, Chang HY (2016). Long Noncoding RNAs in Cancer Pathways. Cancer Cell.

[9] Glud M, Gniadecki R (2013). MicroRNAs in the pathogenesis of malignant melanoma. J Eur Acad Dermatol Venereol.

[10] Tang T, Eldabaje R, Yang L (2016). Current Status of Biological Therapies for the Treatment of Metastatic Melanoma. Anticancer Res.

[11] Gutschner T, Diederichs S (2012). The hallmarks of cancer: a long non-coding RNA point of view. RNA Biol.

[12] Evans JR, Feng FY, Chinnaiyan AM (2016). The bright side of dark matter: lncRNAs in cancer. J Clin Invest.

[13] Lennox KA, Behlke MA (2016). Cellular localization of long non-coding RNAs affects silencing by RNAi more than by antisense oligonucleotides. Nucleic Acids Res.

[14] Li CH, Chen Y (2013). Targeting long non-coding RNAs in cancers: progress and prospects. Int J Biochem Cell Biol.

[15] Gilbert LA, Larson MH, Morsut L, Liu Z, Brar GA, Torres SE, Stern-Ginossar N, Brandman O, Whitehead EH, Doudna JA, Lim WA, Weissman JS, Qi LS (2013). CRISPR-mediated modular RNA-guided regulation of transcription in eukaryotes. Cell.

[16] Ho TT, Zhou N, Huang J, Koirala P, Xu M, Fung R, Wu F, Mo YY (2015). Targeting non-coding RNAs with the CRISPR/Cas9 system in human cell lines. Nucleic Acids Res.

[17] Wang KC, Chang HY (2011). Molecular mechanisms of long noncoding RNAs. Mol Cell.

[18] Rinn JL, Kertesz M, Wang JK, Squazzo SL, Xu X, Brugmann SA, Goodnough LH, Helms JA, Farnham PJ, Segal E, Chang HY (2007). Functional demarcation of active and silent chromatin domains in human HOX loci by noncoding RNAs. Cell.

[19] Wang KC, Yang YW, Liu B, Sanyal A, Corces-Zimmerman R, Chen Y, Lajoie BR, Protacio A, Flynn RA, Gupta RA, Wysocka J, Lei M, Dekker J (2011). A long noncoding RNA maintains active chromatin to coordinate homeotic gene expression. Nature.

[20] Lai F, Orom UA, Cesaroni M, Beringer M, Taatjes DJ, Blobel GA, Shiekhattar R (2013). Activating RNAs associate with Mediator to enhance chromatin architecture and transcription. Nature.

[21] Hu W, Alvarez-Dominguez JR, Lodish HF (2012). Regulation of mammalian cell differentiation by long non-coding RNAs. EMBO Rep.

[22] Batista PJ, Chang HY (2013). Long noncoding RNAs: cellular address codes in development and disease. Cell.

[23] Ma L, Bajic VB, Zhang Z (2013). On the classification of long non-coding RNAs. RNA Biol.

[24] Mukherjee N, Calviello L, Hirsekorn A, de Pretis S, Pelizzola M, Ohler U (2017). Integrative classification of human coding and noncoding genes through RNA metabolism profiles. Nat Struct Mol Biol.

[25] Garbe C, Peris K, Hauschild A, Saiag P, Middleton M, Bastholt L, Grob JJ, Malvehy J, Newton-Bishop J, Stratigos AJ, Pehamberger H, Eggermont AM, European Dermatology F (2016). Diagnosis and treatment of melanoma. European consensus-based interdisciplinary guideline - Update 2016. Eur J Cancer.

[26] Uzdensky AB, Demyanenko SV, Bibov MY (2013). Signal transduction in human cutaneous melanoma and target drugs. Curr Cancer Drug Targets.

[27] Davies H, Bignell GR, Cox C, Stephens P, Edkins S, Clegg S, Teague J, Woffendin H, Garnett MJ, Bottomley W, Davis N, Dicks E, Ewing R (2002). Mutations of the BRAF gene in human cancer. Nature.

[28] Rubinstein JC, Sznol M, Pavlick AC, Ariyan S, Cheng E, Bacchiocchi A, Kluger HM, Narayan D, Halaban R (2010). Incidence of the V600K mutation among melanoma patients with BRAF mutations, and potential therapeutic response to the specific BRAF inhibitor PLX4032. J Transl Med.

[29] Bhatia P, Friedlander P, Zakaria EA, Kandil E (2015). Impact of BRAF mutation status in the prognosis of cutaneous melanoma: an area of ongoing research. Ann Transl Med.

[30] Ugurel S, Rohmel J, Ascierto PA, Flaherty KT, Grob JJ, Hauschild A, Larkin J, Long GV, Lorigan P, McArthur GA, Ribas A, Robert C, Schadendorf D (2016). Survival of patients with advanced metastatic melanoma: The impact of novel therapies. Eur J Cancer.

[31] Flaherty KT, McArthur G (2010). BRAF, a target in melanoma: implications for solid tumor drug development. Cancer.

[32] Wagle N, Emery C, Berger MF, Davis MJ, Sawyer A, Pochanard P, Kehoe SM, Johannessen CM, Macconaill LE, Hahn WC, Meyerson M, Garraway LA (2011). Dissecting therapeutic resistance to RAF inhibition in melanoma by tumor genomic profiling. J Clin Oncol.

[33] Jarkowski A, Khushalani NI (2014). BRAF and beyond: Tailoring strategies for the individual melanoma patient. J Carcinog.

[34] Postow MA, Callahan MK, Wolchok JD (2015). Immune Checkpoint Blockade in Cancer Therapy. J Clin Oncol.

[35] Ribas A, Hamid O, Daud A, Hodi FS, Wolchok JD, Kefford R, Joshua AM, Patnaik A, Hwu WJ, Weber JS, Gangadhar TC, Hersey P, Dronca R (2016). Association of Pembrolizumab With Tumor Response and Survival Among Patients With Advanced Melanoma. JAMA.

[36] Xie HW, Wu QQ, Zhu B, Chen FJ, Ji L, Li SQ, Wang CM, Tong YS, Tuo L, Wu M, Liu ZH, Lv J, Shi WH (2014). Long noncoding RNA SPRY4-IT1 is upregulated in esophageal squamous cell carcinoma and associated with poor prognosis. Tumour Biol.

[37] Mouraviev V, Lee B, Patel V, Albala D, Johansen TE, Partin A, Ross A, Perera RJ (2016). Clinical prospects of long noncoding RNAs as novel biomarkers and therapeutic targets in prostate cancer. Prostate Cancer Prostatic Dis.

[38] Liu H, Lv Z, Guo E (2015). Knockdown of long noncoding RNA SPRY4-IT1 suppresses glioma cell proliferation, metastasis and epithelial-mesenchymal transition. Int J Clin Exp Pathol.

[39] Xie M, Nie FQ, Sun M, Xia R, Liu YW, Zhou P, De W, Liu XH (2015). Decreased long noncoding RNA SPRY4-IT1 contributing to gastric cancer cell metastasis partly via affecting epithelial-mesenchymal transition. J Transl Med.

[40] Mazar J, Zhao W, Khalil AM, Lee B, Shelley J, Govindarajan SS, Yamamoto F, Ratnam M, Aftab MN, Collins S, Finck BN, Han X, Mattick JS (2014). The functional characterization of long noncoding RNA SPRY4-IT1 in human melanoma cells. Oncotarget.

[41] Khaitan D, Dinger ME, Mazar J, Crawford J, Smith MA, Mattick JS, Perera RJ (2011). The melanoma-upregulated long noncoding RNA SPRY4-IT1 modulates apoptosis and invasion. Cancer Res.

[42] Rigoutsos I, Huynh T, Miranda K, Tsirigos A, McHardy A, Platt D (2006). Short blocks from the noncoding parts of the human genome have instances within nearly all known genes and relate to biological processes. Proc Natl Acad Sci U S A.

[43] Akhbari P, Whitehouse A, Boyne JR (2014). Long non-coding RNAs drive metastatic progression in melanoma (Review). Int J Oncol.

[44] Flockhart RJ, Webster DE, Qu K, Mascarenhas N, Kovalski J, Kretz M, Khavari PA (2012). BRAFV600E remodels the melanocyte transcriptome and induces BANCR to regulate melanoma cell migration. Genome Res.

[45] Li R, Zhang L, Jia L, Duan Y, Li Y, Bao L, Sha N (2014). Long non-coding RNA BANCR promotes proliferation in malignant melanoma by regulating MAPK pathway activation. PLoS One.

[46] Su S, Gao J, Wang T, Wang J, Li H, Wang Z (2015). Long non-coding RNA BANCR regulates growth and metastasis and is associated with poor prognosis in retinoblastoma. Tumour Biol.

[47] Shi Y, Liu Y, Wang J, Jie D, Yun T, Li W, Yan L, Wang K, Feng J (2015). Downregulated Long Noncoding RNA BANCR Promotes the Proliferation of Colorectal Cancer Cells via Downregualtion of p21 Expression. PLoS One.

[48] Li L, Zhang L, Zhang Y, Zhou F (2015). Increased expression of LncRNA BANCR is associated with clinical progression and poor prognosis in gastric cancer. Biomed Pharmacother.

[49] Sun M, Liu XH, Wang KM, Nie FQ, Kong R, Yang JS, Xia R, Xu TP, Jin FY, Liu ZJ, Chen JF, Zhang EB, De W (2014). Downregulation of BRAF activated non-coding RNA is associated with poor prognosis for non-small cell lung cancer and promotes metastasis by affecting epithelial-mesenchymal transition. Mol Cancer.

[50] Sousa JF, Torrieri R, Silva RR, Pereira CG, Valente V, Torrieri E, Peronni KC, Martins W, Muto N, Francisco G, Brohem CA, Carlotti CG, Maria-Engler SS (2010). Novel primate-specific genes, RMEL 1, 2 and 3, with highly restricted expression in melanoma, assessed by new data mining tool. PLoS One.

[51] Goedert L, Pereira CG, Roszik J, Placa JR, Cardoso C, Chen G, Deng W, Yennu-Nanda VG, Silva WA, Davies MA, Espreafico EM (2016). RMEL3, a novel BRAFV600E-associated long noncoding RNA, is required for MAPK and PI3K signaling in melanoma. Oncotarget.

[52] Pasmant E, Laurendeau I, Heron D, Vidaud M, Vidaud D, Bieche I (2007). Characterization of a germ-line deletion, including the entire INK4/ARF locus, in a melanoma-neural system tumor family: identification of ANRIL, an antisense noncoding RNA whose expression coclusters with ARF. Cancer Res.

[53] Sarkar D, Leung EY, Baguley BC, Finlay GJ, Askarian-Amiri ME (2015). Epigenetic regulation in human melanoma: past and future. Epigenetics.

[54] Read J, Wadt KA, Hayward NK (2016). Melanoma genetics. J Med Genet.

[55] Yap KL, Li S, Munoz-Cabello AM, Raguz S, Zeng L, Mujtaba S, Gil J, Walsh MJ, Zhou MM (2010). Molecular interplay of the noncoding RNA ANRIL and methylated histone H3 lysine 27 by polycomb CBX7 in transcriptional silencing of INK4a. Mol Cell.

[56] Sun Y, Zheng ZP, Li H, Zhang HQ, Ma FQ (2016). ANRIL is associated with the survival rate of patients with colorectal cancer, and affects cell migration and invasion in vitro. Mol Med Rep.

[57] Nie FQ, Sun M, Yang JS, Xie M, Xu TP, Xia R, Liu YW, Liu XH, Zhang EB, Lu KH, Shu YQ (2015). Long noncoding RNA ANRIL promotes non-small cell lung cancer cell proliferation and inhibits apoptosis by silencing KLF2 and P21 expression. Mol Cancer Ther.

[58] Leucci E, Vendramin R, Spinazzi M, Laurette P, Fiers M, Wouters J, Radaelli E, Eyckerman S, Leonelli C, Vanderheyden K, Rogiers A, Hermans E, Baatsen P (2016). Melanoma addiction to the long non-coding RNA SAMMSON. Nature.

[59] Leucci E, Coe EA, Marine JC, Vance KW (2016). The emerging role of long non-coding RNAs in cutaneous melanoma. Pigment Cell Melanoma Res.

[60] Wu CF, Tan GH, Ma CC, Li L (2013). The non-coding RNA llme23 drives the malignant property of human melanoma cells. J Genet Genomics.

[61] Wang F, Li X, Xie X, Zhao L, Chen W (2008). UCA1, a non-protein-coding RNA up-regulated in bladder carcinoma and embryo, influencing cell growth and promoting invasion. FEBS Lett.

[62] Tian Y, Zhang X, Hao Y, Fang Z, He Y (2014). Potential roles of abnormally expressed long noncoding RNA UCA1 and Malat-1 in metastasis of melanoma. Melanoma Res.

[63] Wei Y, Sun Q, Zhao L, Wu J, Chen X, Wang Y, Zang W, Zhao G (2016). LncRNA UCA1-miR-507-FOXM1 axis is involved in cell proliferation, invasion and G0/G1 cell cycle arrest in melanoma. Med Oncol.

[64] Ji P, Diederichs S, Wang W, Boing S, Metzger R, Schneider PM, Tidow N, Brandt B, Buerger H, Bulk E, Thomas M, Berdel WE, Serve H (2003). MALAT-1, a novel noncoding RNA, and thymosin beta4 predict metastasis and survival in early-stage non-small cell lung cancer. Oncogene.

[65] Gutschner T, Hammerle M, Diederichs S (2013). MALAT1 -- a paradigm for long noncoding RNA function in cancer. J Mol Med (Berl).

[66] Serghiou S, Kyriakopoulou A, Ioannidis JP (2016). Long noncoding RNAs as novel predictors of survival in human cancer: a systematic review and meta-analysis. Mol Cancer.

[67] Sun Y, Cheng H, Wang G, Yu G, Zhang D, Wang Y, Fan W, Yang W (2017). Deregulation of miR-183 promotes melanoma development via lncRNA MALAT1 regulation and ITGB1 signal activation. Oncotarget.

[68] Wu L, Murat P, Matak-Vinkovic D, Murrell A, Balasubramanian S (2013). Binding interactions between long noncoding RNA HOTAIR and PRC2 proteins. Biochemistry.

[69] Gupta RA, Shah N, Wang KC, Kim J, Horlings HM, Wong DJ, Tsai MC, Hung T, Argani P, Rinn JL, Wang Y, Brzoska P, Kong B (2010). Long non-coding RNA HOTAIR reprograms chromatin state to promote cancer metastasis. Nature.

[70] Tang L, Zhang W, Su B, Yu B (2013). Long noncoding RNA HOTAIR is associated with motility, invasion, and metastatic potential of metastatic melanoma. Biomed Res Int.

[71] Bhan A, Mandal SS (2015). LncRNA HOTAIR: A master regulator of chromatin dynamics and cancer. Biochim Biophys Acta.

[72] Cantile M, Scognamiglio G, Marra L, Aquino G, Botti C, Falcone MR, Malzone MG, Liguori G, Di Bonito M, Franco R, Ascierto PA, Botti G (2017). HOTAIR Role in Melanoma Progression and its Identification in the Blood of Patients with Advanced Disease. J Cell Physiol.

[73] Kim HJ, Lee DW, Yim GW, Nam EJ, Kim S, Kim SW, Kim YT (2015). Long non-coding RNA HOTAIR is associated with human cervical cancer progression. Int J Oncol.

[74] Kim K, Jutooru I, Chadalapaka G, Johnson G, Frank J, Burghardt R, Kim S, Safe S (2013). HOTAIR is a negative prognostic factor and exhibits pro-oncogenic activity in pancreatic cancer. Oncogene.

[75] Schmidt K, Joyce CE, Buquicchio F, Brown A, Ritz J, Distel RJ, Yoon CH, Novina CD (2016). The lncRNA SLNCR1 Mediates Melanoma Invasion through a Conserved SRA1-like Region. Cell Rep.

[76] Shi X, Ma C, Zhu Q, Yuan D, Sun M, Gu X, Wu G, Lv T, Song Y (2016). Upregulation of long intergenic noncoding RNA 00673 promotes tumor proliferation via LSD1 interaction and repression of NCALD in non-small-cell lung cancer. Oncotarget.

[77] Aftab MN, Dinger ME, Perera RJ (2014). The role of microRNAs and long non-coding RNAs in the pathology, diagnosis, and management of melanoma. Arch Biochem Biophys.

[78] Smedley D, Sidhar S, Birdsall S, Bennett D, Herlyn M, Cooper C, Shipley J (2000). Characterization of chromosome 1 abnormalities in malignant melanomas. Genes Chromosomes Cancer.

[79] Thorenoor N, Faltejskova-Vychytilova P, Hombach S, Mlcochova J, Kretz M, Svoboda M, Slaby O (2016). Long non-coding RNA ZFAS1 interacts with CDK1 and is involved in p53-dependent cell cycle control and apoptosis in colorectal cancer. Oncotarget.

[80] Chen L, Yang H, Xiao Y, Tang X, Li Y, Han Q, Fu J, Yang Y, Zhu Y (2016). Lentiviral-mediated overexpression of long non-coding RNA GAS5 reduces invasion by mediating MMP2 expression and activity in human melanoma cells. Int J Oncol.

[81] Cao S, Liu W, Li F, Zhao W, Qin C (2014). Decreased expression of lncRNA GAS5 predicts a poor prognosis in cervical cancer. Int J Clin Exp Pathol.

[82] Tu ZQ, Li RJ, Mei JZ, Li XH (2014). Down-regulation of long non-coding RNA GAS5 is associated with the prognosis of hepatocellular carcinoma. Int J Clin Exp Pathol.

[83] Zhao L, Guo H, Zhou B, Feng J, Li Y, Han T, Liu L, Li L, Zhang S, Liu Y, Shi J, Zheng D (2016). Long non-coding RNA SNHG5 suppresses gastric cancer progression by trapping MTA2 in the cytosol. Oncogene.

[84] Lessard L, Liu M, Marzese DM, Wang H, Chong K, Kawas N, Donovan NC, Kiyohara E, Hsu S, Nelson N, Izraely S, Sagi-Assif O, Witz IP (2015). The CASC15 Long Intergenic Noncoding RNA Locus Is Involved in Melanoma Progression and Phenotype Switching. J Invest Dermatol.

[85] Russell MR, Penikis A, Oldridge DA, Alvarez-Dominguez JR, McDaniel L, Diamond M, Padovan O, Raman P, Li Y, Wei JS, Zhang S, Gnanchandran J, Seeger R (2015). CASC15-S Is a Tumor Suppressor lncRNA at the 6p22 Neuroblastoma Susceptibility Locus. Cancer Res.

[86] Spizzo R, Nicoloso MS, Croce CM, Calin GA (2009). SnapShot: MicroRNAs in Cancer. Cell.

[87] Shain AH, Bastian BC (2016). From melanocytes to melanomas. Nat Rev Cancer.

[88] Xu S, Wang H, Pan H, Shi Y, Li T, Ge S, Jia R, Zhang H, Fan X (2016). ANRIL lncRNA triggers efficient therapeutic efficacy by reprogramming the aberrant INK4-hub in melanoma. Cancer Lett.

[89] Xie H, Rachakonda PS, Heidenreich B, Nagore E, Sucker A, Hemminki K, Schadendorf D, Kumar R (2016). Mapping of deletion breakpoints at the CDKN2A locus in melanoma: detection of MTAP-ANRIL fusion transcripts. Oncotarget.

[90] Ichigozaki Y, Fukushima S, Jinnin M, Miyashita A, Nakahara S, Tokuzumi A, Yamashita J, Kajihara I, Aoi J, Masuguchi S, Zhongzhi W, Ihn H (2016). Serum long non-coding RNA, snoRNA host gene 5 level as a new tumor marker of malignant melanoma. Exp Dermatol.

[91] Liu T, Shen SK, Xiong JG, Xu Y, Zhang HQ, Liu HJ, Lu ZG (2016). Clinical significance of long noncoding RNA SPRY4-IT1 in melanoma patients. FEBS Open Bio.

[92] Zhao W, Mazar J, Lee B, Sawada J, Li JL, Shelley J, Govindarajan S, Towler D, Mattick JS, Komatsu M, Dinger ME, Perera RJ (2016). The Long Noncoding RNA SPRIGHTLY Regulates Cell Proliferation in Primary Human Melanocytes. J Invest Dermatol.

[93] Chen L, Yang H, Xiao Y, Tang X, Li Y, Han Q, Fu J, Yang Y, Zhu Y (2016). LncRNA GAS5 is a critical regulator of metastasis phenotype of melanoma cells and inhibits tumor growth in vivo. Onco Targets Ther.

[94] Shi H, Liu L, Liu L, Geng J, Zhou Y, Chen L (2014). beta-Elemene inhibits the metastasis of B16F10 melanoma cells by downregulation of the expression of uPA, uPAR, MMP-2, and MMP-9. Melanoma Res.

[95] Toschi E, Rota R, Antonini A, Melillo G, Capogrossi MC (2000). Wild-type p53 gene transfer inhibits invasion and reduces matrix metalloproteinase-2 levels in p53-mutated human melanoma cells. J Invest Dermatol.

[96] Laxman B, Morris DS, Yu J, Siddiqui J, Cao J, Mehra R, Lonigro RJ, Tsodikov A, Wei JT, Tomlins SA, Chinnaiyan AM (2008). A first-generation multiplex biomarker analysis of urine for the early detection of prostate cancer. Cancer Res.

[97] McCarthy N (2012). Epigenetics. Going places with BANCR. Nat Rev Cancer.

